# An Integrated Multi-Sensor Approach for the Remote Monitoring of Parkinson’s Disease

**DOI:** 10.3390/s19214764

**Published:** 2019-11-02

**Authors:** Giovanni Albani, Claudia Ferraris, Roberto Nerino, Antonio Chimienti, Giuseppe Pettiti, Federico Parisi, Gianluigi Ferrari, Nicola Cau, Veronica Cimolin, Corrado Azzaro, Lorenzo Priano, Alessandro Mauro

**Affiliations:** 1 Istituto Auxologico Italiano, IRCCS, Department of Neurology and NeuroRehabilitation, S. Giuseppe Hospital, 28824 Piancavallo, Oggebbio (Verbania), Italy; g.albani@auxologico.it (G.A.); n.cau@auxologico.it (N.C.); c.azzaro@auxologico.it (C.A.); lorenzo.priano@unito.it (L.P.); alessandro.mauro@unito.it (A.M.); 2 Institute of Electronics, Computer and Telecommunication Engineering, National Research Council, Corso Duca degli Abruzzi 24, 10129 Torino, Italy; claudia.ferraris@ieiit.cnr.it (C.F.); antonio.chimienti@ieiit.cnr.it (A.C.); giuseppe.pettiti@ieiit.cnr.it (G.P.); 3 Department of Neurosciences, University of Turin, Via Cherasco 15, 10100 Torino, Italy; 4 CNIT Research Unit of Parma and Department of Information Engineering, University of Parma, 43124 Parma, Italy; federico.parisi@unipr.it (F.P.); gianluigi.ferrari@unipr.it (G.F.); 5 Department of Electronics, Information and Bioengineering, Politecnico di Milano, 20133 Milano, Italy; veronica.cimolin@polimi.it

**Keywords:** Parkinson’s disease, UPDRS assessment, RGB-depth cameras, body sensor networks, hand tracking, human machine interface, machine learning, remote monitoring

## Abstract

The increment of the prevalence of neurological diseases due to the trend in population aging demands for new strategies in disease management. In Parkinson’s disease (PD), these strategies should aim at improving diagnosis accuracy and frequency of the clinical follow-up by means of decentralized cost-effective solutions. In this context, a system suitable for the remote monitoring of PD subjects is presented. It consists of the integration of two approaches investigated in our previous works, each one appropriate for the movement analysis of specific parts of the body: low-cost optical devices for the upper limbs and wearable sensors for the lower ones. The system performs the automated assessments of six motor tasks of the unified Parkinson’s disease rating scale, and it is equipped with a gesture-based human machine interface designed to facilitate the user interaction and the system management. The usability of the system has been evaluated by means of standard questionnaires, and the accuracy of the automated assessment has been verified experimentally. The results demonstrate that the proposed solution represents a substantial improvement in PD assessment respect to the former two approaches treated separately, and a new example of an accurate, feasible and cost-effective mean for the decentralized management of PD.

## 1. Introduction

In 2030, in Western Europe and in the ten most populous nations of the world, the estimated number of individuals with Parkinson’s disease (PD) will be over 8 million [1]. One of the main challenges to be faced will be the development of technological and cost-effective solutions to improve the healthcare of these patients. Remote medical communications, for example in the form of telemedicine and decentralized care pathways, will inevitably become part of the clinical management of such disease.

Among the tougher hurdles to overcome, there is the accurate, low-cost and objective quantification of the typical motor symptoms, which is a key issue for obtaining a complete indication of the patient’s state of impairment. Since 1967, when the motor effects of Levodopa (LD) on bradykinesia have been shown for the first time in a video [2], the way has been traced towards the quantification of movement disorders and their therapeutic response. This pathway passes through semi-quantitative rating scales, such as the Hoehn and Yahr stage and unified Parkinson’s disease rating scale (UPDRS), which is still the gold standard scale for assessing symptoms in PD [3]. Evaluation through the UPDRS score is a subjective process that comes after a global clinical judgment for the diagnosis in which the experience of the specialist in movement disorders is crucial. This process is, in fact, characterized by an intrinsic inter-rater variability due to the different experience of the examiners, influencing the final judgment on the impairment severity [4]. A percentage of incorrect diagnosis of 25% has also been reported, especially when symptoms such as essential tremor, vascular parkinsonism and atypical parkinsonian syndromes manifest [5]. 

Another difficulty to overcome, shared by all clinical assessment scales, is the mandatory presence of the specialist: this strongly influences the clinical management of the patient, as all the therapeutic decisions are restricted at the time of the single medical examination. However, during the time interval between two consecutive examinations, the patient’s motor status may change significantly, out of the examiner’s sight. The chance of monitoring any change of the impairment in PD subjects over time, possibly daily and in a domestic environment, could have a strong impact on the clinical management of the patients and, consequently, on the long-term costs for the healthcare system.

In an attempt to address these challenges and quantify motor symptoms, motion analysis studies have played a growing role in managing subjects with PD, exploring kinematic variables on gait [6,7]; tremor [8,9]; bradykinesia [10,11] and rigidity [12,13]. Motion capture systems are the reference standard for motion analysis, but they are expensive and non-portable, so their use is limited to laboratory environments and scientific research rather than to a routine clinical use. 

Therefore, in order to overcome these hurdles, it is urgent to find automatic and cost-effective solutions, capable of providing accurate and objective kinematic measures of motor performance and automatic scores that are well correlated to the standard clinical ones [14].

Recently, various technological approaches have been proposed for the analysis of movement in PD [15], including wearable sensor networks [16,17,18,19,20,21] based on the use of inertial measurement units (IMUs), smartphones [22] and active vision systems [23,24,25] based on new low-cost RGB-Depth optical devices such as Microsoft Kinect^®^ (Microsoft Corporation, Redmond, WA, USA) and Intel RealSense^®^ SR300 (Intel Corporation, Santa Clara, CA, USA)[26].

On the basis of these considerations, we propose the integration of two different sensor-based subsystems as a single solution for the objective, accurate and automated assessment of some standard tasks for the upper and lower limbs, as defined in the motor examination section (Section 3) of UPDRS. The first component is an active vision system dedicated to the analysis of the motor tasks related to upper limbs; the second applies a body sensor network (BSN) approach with wireless wearable sensors for the analysis of the lower limb tasks. The technical details of both and the validation of their accuracy as UPDRS automatic scoring systems have been already presented in previous works [27,28,29,30] and will be briefly summarized in the Materials and Methods section. 

A gesture-based human machine interface has been developed as part of the solution, to simplify the interaction and the self-management of the system by people with impairment, in view of a possible use at home.

The system is able to analyze and automatically score the patient movements during the performance of UPDRS tasks. In addition, it archives in the Cloud the kinematic data, the score and the video of each motor performance, guaranteeing the remote supervision by clinicians and overcoming the mandatory presence of the specialist during the execution of the motor activities. Finally, in view of a decentralized approach to the clinical management of the patient, the issue of acceptability and usability of the integrated system has been evaluated through a standard questionnaire.

The integrated system here presented is aimed at a more comprehensive evaluation of the neuro-motor status of PD subjects by a decentralized approach, and could represent a new example of an accurate, feasible and cost-effective strategy for the clinical management of PD.

## 2. Materials and Methods

The integrated monitoring system is designed for the remote, automated and quantitative assessment of six standard tasks defined in the motor examination section of the UPDRS, namely: finger tapping (FT), pronation–supination (PS), hand closing–opening (CO), leg agility (LA), sit to stand (S2S) and gait (G).

The automated assessment is obtained by analyzing the movement of upper and lower limbs during the execution of the aforementioned UPDRS tasks. Specific kinematic parameters are then automatically extracted from the signals representing the subject’s hand and body movements. Afterwards, these parameters are used by the system to predict the UPDRS score assigned to the motor performance. A machine learning approach based on trained supervised classifiers, one for each task, is used for the automated assessment. The motion tracking for the upper limbs is performed by the active vision subsystem based on RGB-depth optical devices, while the BSN subsystem, based on wearable wireless inertial sensors, is used for tracking the movement of trunk and lower limbs.

The integration of the two subsystems exploits the advantages of both approaches: a non-invasive tracking for fine hand movements by optical devices; an occlusion-free tracking for coarser lower limb movements by wearable sensors. The advantage of combining technologies based on different sensors is to have several simple and effective solutions suitable for the most accurate analysis of each single motor task and usable for the future extension to other tasks and domains of interest.

We point out that the integrated approach is not just the superimposition of the two subsystems developed in our previous works: the challenge was to verify if the system performs better than the separate solutions, not only in terms of number of analyzed UPDRS tasks but as a whole integrated solution, both in terms of usability and evaluation accuracy. Moreover, the integration of more tasks into a single evaluation platform could permit to obtain the UPDRS scoring, both for a single task and for group of tasks, in few minutes, reproducing how the UPDRS scoring is actually obtained by clinicians. For this purpose, we designed an experiment to assess simultaneously, both by the system and by clinicians, new groups of healthy control (HC) and PD subjects, which are different from those used for training the supervised classifiers of the original subsystems. In fact, the classifiers used in this work were trained with the data collected in our previous works. Furthermore, the integrated system makes use of a gesture-based human machine interface (HMI) designed for people with motor impairment and used to simplify the interaction and the self-management of the system in a domestic environment. Before starting the experimental evaluation of the system performance, the subjects performed a training phase to get them acquainted with the system interfaces for the self-management of the UPDRS tests. The details of the two subsystems, the description of the human machine interface, the evaluation of the accuracy and usability of the integrated system are described in the following subsections.

### 2.1. Description of the Integrated System 

#### 2.1.1. Upper Limbs Subsystem

**Figure 1:**
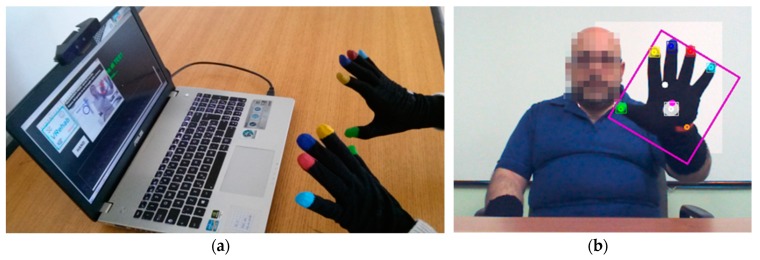
The upper limbs subsystem. (**a**) Example of configuration with notebook for the analysis of fine hand movements and (**b**) tracking algorithm at work: tracking of hand and fingers movements through detection and tracking of 3D-colored blob centroids.

A low-cost tracking system has been developed for the accurate acquisition of fine hand movements [29,30] (Figure 1a): it consists of an RGB-depth camera (Intel RealSense^®^ SR300 [26]), a computer (notebook or mini-PC), a monitor (for visual feedback of the hand movement) and easy-to-wear gloves with colored markers imprinted on specific parts of the hand. 

The RGB-depth camera produces, through its Software Development Kit (SDK), synchronized color and depth streams at about 60 frame/sec, which is suitable for the real-time acquisition of the hand movement. Subjects perform the upper limb tasks of the UPDRS (i.e., FT, CO and PS) comfortably seated in front of the camera wearing the gloves. The dedicated tracking algorithms, based on computer vision techniques, detect the colored markers in the scene and calculate the three-dimensional (3D) trajectories of each colored blob centroid, thus capturing the movements of hand and fingers (Figure 1b). The fusion of color and depth information performed by the tracking algorithms ensures greater robustness compared to the different commercial trackers based only on depth information, as shown by comparison with gold reference systems for motion analysis [29]. This approach also acts as a gesture-based HMI, making the interaction with the system friendly, self-manageable and natural [30]. The initial setup of the subsystem consists of a calibration procedure in which the subject is asked to keep one hand raised and open in front of the camera. In this way, the subsystem automatically determines the ambient lighting to correctly set the internal thresholds for the detection and the color adjustment. This procedure is used to adapt the tracking to the environmental conditions.

The representative kinematic parameters, used for the characterization of the upper limb movements and the automatic assessment in the form of UPDRS score, are 10 for FT, 8 for CO and 8 for PS, respectively (Table S1 in Supplementary Materials for the list of parameters and their meaning). They are extracted from the 3D trajectories and consist of the same physical quantities (e.g., amplitude, speed, rhythm, regularity and anomalies) implicitly considered by neurologists for the standard clinical assessment of motor performance, as indicated by UPDRS guidelines for the assessment of each task.

#### 2.1.2. Lower Limbs Subsystem

**Figure 2:**
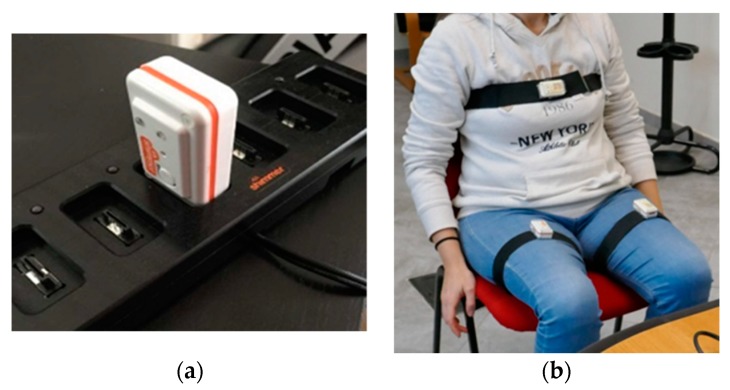
The lower limbs subsystem. (**a**) An inertial measurement unit (IMU) wireless sensor in the battery charger and (**b**) the designed configuration, with one sensor per thigh and one on the chest.

A BSN tracking system was developed to acquire the movements of trunk and lower limbs: it consisted of a series of low-power commercial wireless IMUs (Shimmer^®^, Shimmer Research, Cambridge, MA, USA). Each node was equipped with a tri-axial accelerometer, magnetometer and gyroscope [27], thus a total of 9 degrees of freedom (Figure 2a). The sampling rate was 100 samples/sec: the stream of data acquired was sent via Bluetooth connection to the same computer (notebook or mini-PC) used for the upper limbs.

The designed configuration consisted of two sensors on the thighs (one per thigh) and one on the chest (Figure 2b), positioned on subject’s body using special straps to maintain the correct position of the nodes during the execution of the lower limb tasks of the UPDRS (i.e., LA, S2S and G). The positioning of the nodes was chosen considering the need to analyze the three tasks without changing the configuration of the sensors, in order to reduce the subject’s stress and to simplify the acquisition procedure. Before starting the acquisition, it was necessary to carry out a guided calibration procedure to check both the correct alignment of the sensors and the Bluetooth connection of all the nodes: in case of incorrect alignment, a warning message is displayed on the monitor until the supervisor (such as the examiner or the caregiver) corrects the position of the node.

Dedicated signal processing algorithms allows us to capture the real-time kinematic parameters of the movements of trunk and legs during the execution of UPDRS tasks [28]. The sets of representative kinematic parameters, used for the characterization of the lower limb movements and the automatic assessment in the form of UPDRS scores, were seven for LA, six for S2S and nine for G, respectively (Table S1 in Supplementary Materials for the parameter list and their meaning). Parameters were extracted from the inclination and angular velocities: they correspond to the same physical quantities taken into account by neurologists to assess the motor performance, according to the UPDRS guidelines.

### 2.2. Gesture-Based Human Machine Interface

The gesture-based HMI is designed for users with motor impairment to simplify system interaction and self- management, especially for applications in home environment. At startup, it is assumed that the user is in front of the RGB-D camera and is able to see the system monitor. A graphical user interface (GUI) menu prompts to choose between upper or lower limb tasks. The user makes the choice by raising the left or right hand. The depth stream provided by the camera is processed by specific algorithms to detect significant variations in the depth map on one of the user’s sides, thus establishing the user’s selection.

**Figure 3:**
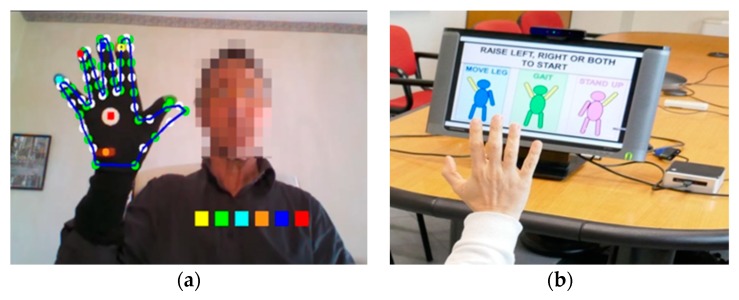
Example of the human machine interface. (**a**) Graphical user interface (GUI) for color calibration procedure (upper limb subsystem) and (**b**) GUI for task selection (lower limb subsystem).

If the tasks for the upper limbs are selected, a new menu is presented by the GUI on the monitor concerning which task (FT, CO or PS) has to be performed. In this case, the user’s choice is made by shifting the hand on one of the selection boxes proposed by the GUI, and it is acknowledged by closing the gloved hand. Subsequently, the user is guided by text messages on the GUI in the interaction with the system to complete some operating procedures, such as the automated initial color calibration (Figure 3a), and then to perform the motor task selected. During the execution of the task, the subsystem acquires video and kinematic data; then it automatically evaluates the motor performance by assigning a predictive score ([30] for details).

In the case of the selection of lower limb tasks, a new selection GUI was proposed on the monitor, concerning which task (LA, S2S and G) had to be performed. This time, the user choice was recognized by raising the left, the right or both hands, as shown in Figure 3b. For lower limb tasks, the start of the task execution could not be managed directly by the upper limb subsystem since the motor performance was carried out far from the RGB-D camera to ensure also the video acquisition of the full body movements for an eventual remote supervision. Therefore, in this case the beginning of the task execution was given by a sharp blow of the heel, which was detected in the acceleration signal of the IMU placed on the leg.

### 2.3. Description of the Experimental Framework

#### 2.3.1. Participant Recruitment

The accuracy of the system in the assessment of motor tasks was evaluated by an experiment conducted on two groups of subjects, the first consisting of healthy controls (HC) and the other of patients with PD. The HC group was included in the experimental test both to analyze the discriminatory power of the system in distinguishing healthy from PD subjects, and to normalize the kinematic parameters of the PD group by the corresponding average values of the HC subjects’ parameters. This was made to avoid bias in the results due to the different ranges of the parameter values (refer to the data analysis subsection). 

The PD group was composed of 25 subjects with PD (15 males and 10 females; mean values: age, 66.9 years; disease duration, 7.5 years; Hoehn and Yahr score, 2.5). PD subjects were excluded if they had: a history of neurosurgical procedures, tremor severity >1 (according to UPDRS-III severity score) or cognitive impairment (mini-mental state examination score <27/30). All PD subjects were allowed to take their routine medications. The HC group was composed of 15 volunteers (nine males and six females; mean value: age, 66.4 years). HC subjects were excluded if they had any motor, neurological or cognitive impairment. Informed consent was obtained from the participants. The Ethics Committee of the Istituto Auxologico Italiano approved this study (Protocol n. 2011_09_27_05).

#### 2.3.2. Acquisition and Evaluation Protocol

The motor performances of the subjects, for each of the six UPDRS tasks, were evaluated simultaneously in the outpatient setting both by the system and by a neurologist (indicated as N1) expert in movement disorders. The neurologist assigned the UPDRS score to each single task as required by the UPDRS guidelines. It should be noted that the severity class is a concept that does not define the patient but the motor performance of the patient in the execution of a specific motor task. Therefore, the same subject can belong to different severity classes, with respect to different tasks. This also means that the severity class is not an absolute but a punctual judgment, linked to the single motor performance and the moment in which the patient is assessed. As previously indicated, at the same time the video of each performance was recorded by the system to allow possible actions of remote supervision. Each task was performed according to the UPDRS guidelines [3]. Each PD subject was evaluated during the “on” phase, approximately 1 h after taking the L-Dopa dose according to the individual pharmacological treatment. Before starting the test session, randomization was applied to establish the order of the sequence of tasks to be performed. Each HC subject was asked to perform the same motor tasks in the same environmental conditions as the PD subjects, in order to relate the motor performance of the PD group to the HC group, providing normalized values for the kinematic parameters of the PD group.

Three months later, each recorded video of the performance of PD subjects was assessed again by the same neurologist (N1) and by two other experienced colleagues (indicated as N2 and N3). This activity was carried out to estimate the inter-rater variability among the specialists, the intra-rater variability over time and to evaluate the possibility of using the video recording for the remote performance assessment, avoiding the presence of a specialist during the execution of the motor task.

### 2.4. Data Analysis and Statistics

The analysis of the data addressed two main questions: the evaluation of the accuracy of the integrated system in the assignment of the correct automatic score to the subject’s performance with respect to the clinical scores, and the reliability of the remote assessment of the subject’s performance by the specialists based on the videos recorded.

Before being processed, the kinematic parameters of the PD subjects were normalized on the basis of the corresponding average values of parameters of the HC subjects, in order to avoid bias in the results only due to the different range of values and not to real clinical aspects. In particular, the PD parameters (*p*_i PD_) have been normalized by the corresponding mean values of parameters for the HC subjects (*p*_i HCMean_) according to the following formula (Equation (1)), which has been used also in our former works. The formula scales the parameter values, which represent different physical quantities, into comparable excursion ranges. Furthermore, it takes into account that, on average, the kinematic parameters of HC are better (greater) than those of PD and, therefore, they can be used as reference values.

*p*_i PD Norm_ = *p*_i PD_/*p*_i HCMean._ (1)

The effectiveness of the system in the correct classification of HC and the PD subjects, whose motor performance were assessed in four UPDRS severity classes from 0 (no impairment) to 3 (moderate impairment), was evaluated preliminarily by analyzing the discriminatory power of each kinematic parameter used by the supervised classifiers. The ability to discriminate these categories was evaluated by the consistency and the separation among classes shown by the mean values of the kinematic parameters for the HC and PD subjects, this for each of the six UPDRS tasks examined.

Looking at telemonitoring applications and at possible remote supervision tasks, in which the specialist assess the performance only by the recorded video, the agreement between “live”, “video” and “instrumental” assessments is an important factor to consider, which has been addressed by using the intra class correlation (ICC) [31] and the specific ICC model according to the case under examination [32].

First of all, the ICC_N1-SY_ inter-rater reliability (two-way random effects model with an absolute agreement [32]) between “live” and “instrumental” scores was evaluated to measure the agreement between the “live” clinical scores assigned by the reference neurologist (N1) at the end of each patient’s performance and the “instrumental” automatic scores assigned, simultaneously, by the system (SY). Secondly, to verify if the recorded videos (V) of the patients’ performance convey enough information to allow a reliable assessment in remote supervision mode, the ICC_N1-V_ intra-rater reliability (two-way mixed effects model for absolute agreement) between the “live” and the “video” scores assigned by N1 was evaluated [33]. This approach is justified by previous results on the reliability of UPDRS assessments based on videotapes [34]. In practice, N1 reviewed all patients’ performance from recorded videos three months later to rule out any memory on his new assigned scores. Thirdly, to avoid bias in judgments due to a single rater (N1), all recorded videos of the patients’ performance were assessed in randomized order by two other experienced neurologists (N2 and N3), whose judgments were not influenced by the memory of the live performance. The average inter-rater reliability among the three neurologists ICC_N1, N2,N3-V_ (two-way random effects model with an absolute agreement) was considered as a measure of the reliability of the video assessment procedure. During the decision-making process, the three raters also tried to agree on the UPDRS scores assigned to the same patient’s performance but, when impossible, the average value (N123) of the three UPDRS scores was considered as the consensus clinical score, to be compared with the system one. Finally, the inter-rater agreement ICC_N123-SY_ (two-way random effects model with an absolute agreement) between the consensus scores (N123) of neurologists and the “instrumental” automatic scores assigned by system (SY) was evaluated. To evaluate the performance of the system as integrated solution, the agreement between system and clinical assessments as sum of group of tasks was analyzed. In particular, three groups of task were considered: the upper limb tasks (i.e., the sum of the scores assigned to FT, CO and PS for the left and right limbs); the lower limb tasks (i.e., sum of the scores assigned to LA for the left and right limbs, S2S and G); all the six tasks together (i.e., sum of the scores assigned to the performance of all the six tasks examined). In this context, the agreement between the scores assigned by N1 and the system of groups of tasks (ICC_SUM,N1-SY_) was considered, as well as the agreement between the consensus scores (N123) and the system (ICC_SUM,N123-SY_).

The effectiveness of the system in the automatic classification of the patients’ performance was further validated by evaluating the accuracy of each supervised classifier. The accuracy of the classifier, estimated by the confusion matrix, is normally defined as the sum of true positives and true negatives, divided by the number of total instances. In our experiment, multi-class supervised classifiers were used and trained on almost numerically balanced classes: in this case, it seemed more appropriate to use the per-class accuracy, in which the classification accuracy of each class was averaged over all the classes [35]. Several measurements of the classification accuracy were considered for each of the six classifiers (one for task): the accuracy of the system in the correct classification of HC and PD subjects (ACC_HC-PD_) ; the accuracy of the system in the classification of PD subjects with respect to their severity class (i.e., clinical UPDRS score), using the “video” scores of N1 as the clinical reference score (ACC_PDHC,N1-SY_) and, finally, the accuracy of the system in the classification of PD subjects with respect to their severity class, considering the consensus scores (N123) as the clinical reference UPDRS score (ACC_PDHC,N123-SY_).

### 2.5. Questionnaire on Usability of the System 

One of the most important features of a technological solution is the usability, in particular when it has to be used autonomously by people with disabilities and/or little experience in technology, possibly without direct technical supervision. In the context of remote applications, which could become part of the new strategies of disease management in the near future, the good level of usability of a technological solution represents a critical point, as it could influence the results of the self-managed and home-based monitoring of the patient.

To this end, a standard post-study system usability questionnaire (PSSUQ) [36] was presented to each PD participant at the end of the test session. The questionnaire consists of 19-items through which users can express the degree of satisfaction with regard to six main characteristics related to the system usability: ease of use, learnability, effectiveness, simplicity, adequacy and availability of information and feeling about the user interface. As a matter of fact, PSSUQ allows for a more detailed analysis of system usability compared to other questionnaires, since the items can be grouped into different categories, namely system usefulness (questions 1–8), information quality (questions 9–15), interface quality (questions 16–18) and overall judgment (question 19). Each subject was asked to assign an ordinal score to each question, based on a 7-point Likert scale, where one corresponds to “totally disagree” and seven corresponds to “totally agree”. As a consequence, higher average scores on the 19-items indicate greater overall user satisfaction. The 19-items of the PSSUQ are included in Table S2 of Supplementary Materials. Considering that the solution consists of two subsystems characterized by different operating modes, some items of the PSSUQ (in particular, items 6 and 7, which refer specifically to the use of the instrumentation; and items 16 and 17, which consider the instrumentation as part of the user interface and human-machine interaction) were also proposed separately for each subsystem, to assess the potential differences of the two sensor-based approaches with respect to the user experience. Finally, to make the statistical analysis more complete, each user was classified according to his/her own computer literacy into one of four categories: none, basic, intermediate and advanced. The four categories were defined considering as an objective as possible abilities (ability to turn on/off the computer and to use the mouse and keyboard) and the computer usage time [37]. In particular, the level “none” was determined by the first question (“Are you able to turn on/off the computer, to use mouse and keyboard?”) if the subject was not able to perform these basic operations. Otherwise, the second question about computer usage time (“How many times, on average, do you use the computer during the week?”) determined the basic (less than twice a week), intermediate (about three or four times a week) and advanced (almost every day) levels respectively. 

## 3. Results

### 3.1. Data Analysis and Statistics

**Figure 4:**
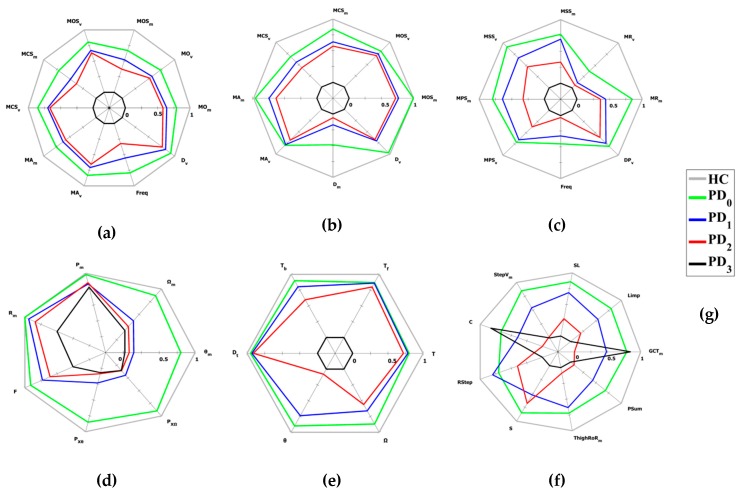
Radar charts of the kinematic parameters for upper and lower limb tasks. Mean values of the most significant kinematic parameters for healthy control (HC) and Parkinson’s disease (PD) subjects, grouped by severity classes according to the “video” scores assigned by N1, decrease monotonically with the increase of the impairment severity (Table S1 in Supplementary Materials for the parameter list and their meaning). (**a**) Finger tapping; (**b**) closing–opening; (**c**) pronation–supination; (**d**) leg agility; (**e**) sit to stand; (**f**) gait and (**g**) legend for HC and PD severity classes (HC refers to healthy controls; PD_0_ refers to UPDRS 0 severity class; PD_1_ refers to UPDRS 1 severity class; PD_2_ refers to UPDRS 2 severity class; PD_3_ refers to UPDRS 3 severity class).

In Figure 4, the average values of the kinematic parameters for the HC and PD subjects involved in the experiment were plotted as radar charts for the six UPDRS tasks. For each motor task, the mean values of the kinematic parameters extracted from the performances of the HC subjects were used as the optimal subset to conveniently scale the kinematic parameters for PD subjects, in order to make them monotonically decreasing in correspondence with the increase of the severity class assigned (i.e., clinical UPDRS score). In this way, all the normalized kinematic parameters were in the range 0–1, where 0 corresponds to the worst value (in general, it should be associated to the most severe UPDRS class) and 1 to the best value (in general, it should be associated to the HC cohort) for each parameter. The radar charts in Figure 4 used the “video” scores assigned by N1 as clinical reference UPDRS scores to aggregate the kinematic parameters of the patients’ performance in the corresponding UPDRS severity classes.

In Table 1 is shown the ICC values for the agreement between N1 “live” scores and the system “instrumental” scores (ICC_N1-SY_); the intra-rater agreement between the “live” and “video” scores assigned by N1 (ICC_N1-V_); the inter-rater agreement among raters (ICC_N1,N2,N3-V_) and the agreement between consensus clinical scores and system scores (ICC_N123-SY_).

According to [32], ICC values below 0.5 indicate poor reliability/agreement; between 0.5 and 0.75 a moderate reliability/agreement; between 0.75 and 0.9 a good reliability/agreement and greater than 0.90 indicate excellent reliability/agreement.

The ICC_N1-V_ indicates a good-to-excellent agreement between the “live” and “video” scores assigned by N1 after three months. The classical, absolute and consistency intra-class correlation coefficients [33] indicate the bias was negligible. Although the correlations between live and video assessments were not excellent, particularly for complex motor tasks, the results indicate that the recorded videos were able to convey enough information to evaluate the patient as in the performance live. The lowest ICC values were associated to PS and G tasks, which were the most challenging tasks to evaluate due to their complexity. For the PS task, this was due to the rapid and alternating movements of the hand that could influence the perception of anomalies. For the G task, this was due to the simultaneous movement of different parts of the body and to the number of variables to be considered. In both cases, this complexity made it more difficult to evaluate these tasks than others even for an expert evaluator: despite this, a good reliability was obtained. The ICC_N1,N2,N3-V_ indicates a good agreement between the raters in all tasks. The lowest ICC values were associated with the tasks of the upper limbs involving fast movements (FT and PS), and of the lower limbs, involving complex movements (G). The ICC_N1-SY_ indicates moderate to almost good reliability (this last for some tasks), between the “live” scores assigned by N1 and the “instrumental” scores assigned by the system as a result of the automatic classification. The lowest ICC values were again associated with PS and G tasks. It should be noted that, when the clinical consensus scores were used, the ICC values (ICC_N123-SY_) increased for all tasks, highlighting the importance of harmonizing the scores of different raters [34].

In Table 2, the ICC values for the agreement between clinical and system assessments of groups of tasks are shown. The agreement was evaluated considering the upper limb tasks (i.e., sum of the scores assigned to FT, CO and PS for the left and right limbs); the lower limb tasks (i.e., sum of the scores assigned to LA for the left and right limbs, S2S and G) and all the six tasks (i.e., sum of the scores assigned to the performance of all the six tasks examined). Both the N1 and the N123 scores were considered as reference clinical scores.

To evaluate the accuracy of the system in the automatic assessment of the subjects’ performance, two types of classification problems were addressed: a binary classification problem, in which the subjects had to be classified as HC or PD (i.e., two classes); a multi-class classification problem, in which the subjects had to be classified as HC or as one of the four UPDRS severity classes (i.e., five classes). Table 3 shows the results of the classification accuracies for the six tasks. The ACC_HC-PD_ represents the accuracy of the system in the classification of subjects as HC or PDs (2-classes problem); the ACC_PDHC,N1-SY_ represents the accuracy of the system in the classification of subjects as HC or PD, taking into account the severity class according to the “video” score assigned by N1 (5-classes problem); the ACC_PDHC,N123-SY_ represents the accuracy of the system in the classification of subjects as HC or PD, taking into account the severity class according to the consensus clinical score assigned by the neurologists (five-classes problem). 

### 3.2. Questionnaire on Usability of the System

The statistical analysis of the technological skills of the PD cohort has shown that the majority of the subjects had a rather low level of familiarity in the use of technologies. Only 26% of PD participants declared that they had intermediate or advanced technological skills, while the other 74% had only basic or no experience (over 50% indicated none for their level of experience): this confirms the need to make any technological solution as usable as possible. The percentage breakdown of the four levels of technological skills was 9% (advanced), 17% (intermediate), 21% (basic) and 53% (none), respectively.

**Figure 5:**
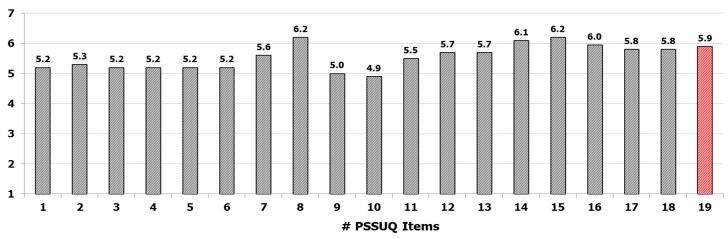
Results of the post-study system usability questionnaire (PSSUQ) on the system usability. The average scores on PD users are indicated for each item. The last column (red) indicates the overall average score assigned to the system and represents the satisfaction of the users. The values for items 6,7,16 and 17 are the average scores assigned separately to upper limbs and lower limbs subsystems.

**Figure 6:**
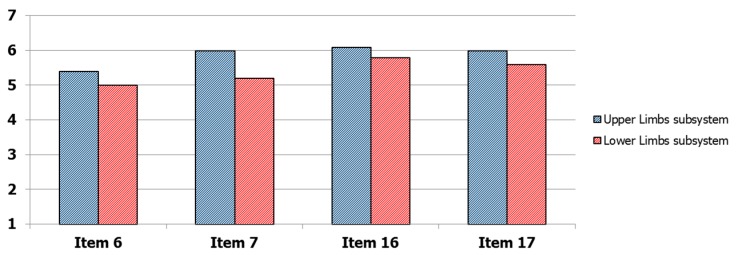
Detail of the mean values of the scores assigned separately for upper limbs subsystem (based on no contact optical devices) and lower limbs subsystem (based on wearable sensors) for the PSSUQ items.

The results on the PSSUQ questionnaire are shown in Figure 5. For each item, the score indicated was averaged on the PD participants. The 19 items were ordered from the first question (on the left) to the last question (on the right). For items 6, 7, 16 and 17 the average scores of the two subsystems were reported. The analysis shows that the participants expressed an overall good satisfaction on their experience in the use of the system: in fact, the average score, calculated on items 1–18, was quite high (items 1–18: 5.5 ± 0.4) as well as the explicit score assigned to item 19 indicating the general level of satisfaction in the use of the system (item 19: 5.9 ± 0.9). Nevertheless, some features of the system have to be improved, for example messages and information to guide the subjects to recover from error conditions (item 9: 5.0 ± 0.8; item 10: 4.9 ± 0.9). However, the results indicate an overall positive judgment by PD participants: in particular, the potential use of this type of solution for the remote monitoring of their health conditions was highly appreciated and expressed through positive free comments. Regarding the differences between the two subsystems in terms of user experience, lower scores were assigned to the subsystem based on wearable sensors, both as technological equipment and as part of the user interface and system interaction, particularly by users with lower technological skills. These differences were visible in Figure 6, where the scores for the items 6, 7, 16 and 17 were shown separately for the two subsystems. Probably, this was due to a greater invasiveness of the subsystem based on wearable sensors compared to the subsystem based on optical device, the less intuitive use and a more complex calibration procedure that requires the execution of specific movements (such as getting up and sitting more times) to properly calibrate the wireless sensors. Nevertheless, the differences were not significant on average, confirming the usability of the wearable sensor subsystem with the adequate training of the end user. 

By grouping the items according to the PSSUQ categories, the analysis indicates that the lowest scores were assigned to the system usefulness category and the highest scores to the interface quality category. This result was closely related to the technological skills of the participants. In fact, the average scores for all PSSUQ categories increased with increasing level of technological skill. Finally, it should be noted that while results for intermediate and advanced subjects were quite similar, there was a significant gap for basic or non-expert users, in particular for the system usefulness category, which was more influenced by the practical management of technological devices. The results of the analysis are shown in Table 4. 

## 4. Discussion

In the perspective of developing new decentralized strategies for the clinical management of PD, it is essential to design technological systems that address some important concerns. First of all, the accurate, objective and consistent characterization of the patient’s performance represents a key point in order to overcome the subjectivity and the intrinsic inter-rater variability of the standard clinical scales such as the UPDRS.

Secondly, the mandatory presence of the specialist for the clinical assessment of the patient’s performance is not feasible in decentralized management approaches.

Thirdly, even if the usability of the technologies is generally considered a secondary aspect in the design, it plays instead a fundamental role, because it can positively or negatively influence the success of remote monitoring applications.

To meet these challenges, we used our previous experience in the development of decentralized monitoring systems for PD. We integrated two previously developed subsystems in a single feasible and cost-effective solution for the objective and automated assessment of six motor tasks of the standard UPDRS scale. The first subsystem is an optical system based on RGB-depth cameras, dedicated to the analysis of the upper limbs tasks; the second one uses a BSN approach based on wireless wearable sensors to analyze the lower limb tasks. The integrated system is equipped with a gesture-based HMI to ensure a simplified interaction for people with motor impairments.

The challenge of the integration was to verify by an experimental campaign on groups of PD and HC subjects the possible improvement achievable respect to the two subsystems, both in terms of usability and accuracy of the assessments.

As regards the objective and accurate characterization of the motor performance, the graphic representation of the average values of the participants’ kinematic parameters, grouped in HC and PD cohorts, confirms the results of the previous works. The graphical representation of the average values, in the form of radar charts, visually indicated that most of the kinematic parameters separate the HC and PD classes distinctly, with no overlapping between the classes and with the HC parameters always larger (better performances) than the PD parameters. At the same time, a gradual reduction of the radar area was evident for the most severe classes as expected, denoting a progressive worsening of the overall performance for the most compromised subjects, as shown in Figure 4. Although this was noticeable for the upper limb tasks, for lower limbs in some cases there was a partial overlap between the intermediate classes, for which the average values of kinematic parameters were quite similar: this could mean that some features of the movement ere not so different for these severity classes (i.e., those parameters were not well related to the severity class). The gait task seemed to be the one with the most complex interpretation, probably due to the involvement of different parts of the body and the presence of various anomalies during the movement that could influence the performance and generate intersections of parameters between intermediate classes. Despite this, also for this task, the results were consistent with the clinical assessment of gait in PD subjects, generally characterized by short steps, irregularities and low speed in the most compromised classes.

The classification accuracies of the supervised classifiers for HC and PD participants reported in Table 3 indicated a similar trend of the ICC values for the six tasks. The accuracies obtained by using the N1 scores (ACC_PDHC, N1-SY_) were worse with respect to the ones obtained by using the consensus clinical scores (ACC_PDHC, N123-SY_) in all tasks. This was expected because the scores assigned by N1 are probably more biased than the consensus clinical scores of the three neurologists. Furthermore, as expected for the same training dataset, the accuracy of the classifiers decreased as the number of classes increased. The accuracy values of the second and third row of Table 3 (multi-class classification) show worse accuracies with respect to the results of the first one for the classification of HC and PD participants (binary classification, considering all PD subjects as a single class). Nevertheless, the ability to classify the PD subject performance in different severity classes is important to assess subtle variations of the disease impairment.

In general, the results on the accuracy of the classifiers confirmed the outcomes of our previous works on different HC and PD cohorts: this is an indication of the consistency in the automated assessment of the motor tasks. Furthermore, the results obtained support the feasibility for a reliable, more accurate and completely automated classification of the severity of disease impairment.

Regarding the results of the reliability/agreement analysis (intra-rater, inter-rater and rater-system), the ICC_N1-SY_ values indicate a moderate agreement between the system scores and the “live” scores of N1, for all the tasks, and between the system and the video-based scores of N1, N2 and N3. In the latter case, the values were closer to good agreement, in particular for the FT, CO, LA and S2S tasks, supporting the feasibility of the automated assessment by the system. The values were high for the upper limb and lower limb tasks, with the exception of gait task G, which had the lowest value. This agreed with the reduced discriminating power of the corresponding parameters (Figure 4f), which had a negative impact on the accuracy of the classifier, which, once again, could depend on the complexity of the evaluation process due to the quantity of parameters involved. Furthermore, this could be due to some differences in the PD cohort used for the training phase of classifiers compared to the participants in this experiment, amplified by the bias of a single rater (N1). The ICC_N1-V_ values indicate a good intra-rater agreement between “live” and “video” scores assigned by N1. Moreover, the classical, absolute and consistency intra-class correlation coefficients [33] indicate the bias was negligible, confirming the feasibility of the remote assessment based on videos, despite some differences in the evaluations especially for more complex tasks. This allowed us to overcome the mandatory presence of the specialist during the patient’s performance. Furthermore, the values were higher for upper limb tasks, while the gait task G had the lowest value once again. This could be probably due to the closer and better detailed view of the subjects in the videos of the upper limb tasks with respect to those of the lower limbs. The ICC_N1,N2,N3-V_ values indicate the good agreement between the three specialists, with differences among the tasks comparable to the results available in the literature. The lowest values were associated with the tasks that involve rapid (FT and PS) or complex (G) movements: this suggests that the speed and the complexity of the movement might influence the perception of the execution in a different way, leading to a different assessment of the performance. Finally, the ICC_N123-SY_ values indicate a better reliability between the clinical consensus scores (N123) and the system scores respect to the single rater case (ICC_N1-SY_). This suggests that the harmonization of scores assigned by different raters could improve the overall performance of the system: probably, the same result would have been achieved if the raters had followed a teaching program to uniformly evaluate all the motor tasks defined into the motor examination section of the UPDRS [34]. The clinical consensus scores were presumably more robust and less biased than those of N1, and were probably better suited to the system scores, whose classifiers were trained on a wider variety of PD subjects and neurologists. However, the results indicate that the system assessment was compatible with the inter-rater variability, but more importantly the results indicate that for the assessment of some motor performance it might be possible to overcome the mandatory live presence of the specialist, who could have a supervisory role in case of remote monitoring applications. In addition, the results of Table 2 concerning the agreement between clinicians and system (ICC_SUM,N1-Sy_ and ICC_SUM,N123-SY_) evaluated on groups of tasks indicates that the performance of the automated assessment improved with respect to the single task from moderate/good to good/excellent. Once again the results were better using the clinical consensus scores, suggesting that it was possible to obtain a more accurate assessment of the general status of the patient’s impairment by considering groups of tasks instead of a single task.

Finally, the gesture-based HMI represents an important element of the solution because it allows an easier interaction and self-management of the entire system, especially in view of an autonomous and domestic use. The usability of the system was evaluated by a standard PSSUQ questionnaire. Nevertheless, it emerged the need to pay more attention to some aspects of the interface, in particular to messages and information provided to the user in case of recovery from error conditions. The analysis confirmed a good satisfaction of the participants in using the system. No significant difference emerged in the average scores assigned to the two subsystems for those items closest to the practical use of the devices, indicating that the developed technological solution was perceived as a single system. Furthermore, the average scores, on the total PSSUQ and on the different categories of the questionnaire, increased progressively with the skill level of the participants. There was also a significant gap between experienced users (i.e., intermediate or advanced skills) and users with little experience (i.e., none or basic skills): the latter showed greater difficulties in the practical use of the system (low system usefulness scores) even if the intuitive user interface was positively judged by these users as well. Probably, this gap could be filled by adequate training sessions dedicated to subjects with less skill. 

Further work is however necessary to extend these results to a greater number of users, by evaluating also the effect on usability and the learning curve obtained from intensive and continuous use of the system.

## 5. Conclusions

A self-managed system for the automated assessment of the upper limb and lower limb motor aspects of Parkinson’s disease was presented. The system integrated two previously developed subsystems for the automated assessment of six standard motor tasks defined in the UPDRS scale: a vision system for the upper limb analysis and a wearable sensors-based system for the analysis of lower limbs. The integrated system was equipped with a low-cost HMI, which provides a gesture-based interaction to increase the feasibility of self-management of the task executions and an accurate characterization of the patient movements by selected kinematic parameters. The validity and accuracy of the integrated system in the automated assessment of the six tasks was verified in an experimental campaign on cohorts of PD and HC subjects. The reliability/agreement of the automated assessment was evaluated by the ICC coefficients between the clinical and the system scores and by the classifier accuracies. The validation of the assessment performed on videos recorded by the system during the execution of the tasks made viable the remote supervision of the patient, without the mandatory presence of the specialist during the patient’s performance. The evaluation of the system usability through a standard questionnaire indicates the positive experience of the participants in the autonomous interaction with the system, even if some features have to be improved (such as the message support to recover from error conditions). The current system had also some limitations. The fast-moving market of the RGB-depth sensors used in the upper limb subsystem quickly made these devices obsolete, and new ones must be sought to replace them. The lower limb subsystem required a more complex maintenance and configuration than the upper limb subsystem, making its usability slightly lower, as pointed out in the usability analysis. Finally, the limited size of the current datasets used to train the classifiers, had an impact on the assessment accuracy of the system, and further experiments will be needed to extend the reference databases and obtain possible improvements of the classification performance. Nevertheless, the results show that the presented solution, although made of two different subsystems, exhibited a good accuracy in the automated assessments and it was perceived as quite feasible by the users. These features confirmed that the integrated system could be reliably employed as a self-managed system for automated and remote assessment of Parkinson’s disease. Although more work is needed to consolidate these findings, this solution could be considered an example of a new accurate, feasible and cost-effective strategy for decentralized disease management.

## 6. Patents

System and method for motion capture: US10092220B2 (2018), EP3120294A1 (2014) and WO2015139750A1 (2015)

## Figures and Tables

**Table 1:** Intra class correlation coefficients ^a^ for system reliability and raters’ agreement.

Reliability/Task	FT	CO	PS	LA	S2S	G
ICC_N1-SY_	0.73	0.70	0.65	0.72	0.70	0.55
ICC_N1-V_	0.93	0.95	0.87	0.93	0.95	0.80
ICC_N1,N2,N3-V_	0.76	0.80	0.75	0.80	0.82	0.75
ICC_N123-SY_	0.75	0.72	0.68	0.74	0.73	0.61

a: 95% confidence interval.

**Table 2:** Intra class correlation coefficients ^a^ for system and raters’ agreement (on group of tasks).

Reliability/Task	UPPER LIMB TASKS	LOWER LIMB TASKS	ALL SIX TASKS
ICC_SUM_,_N1-SY_	0.88	0.84	0.90
ICC_SUM_,_N123-SY_	0.90	0.86	0.94

a: 95% confidence interval.

**Table 3:** Classification accuracies for the six tasks.

Accuracy/Task	FT	CO	PS	LA	S2S	G
ACC_HC-PD_	98.3	92.4	98.6	92.5	91.5	93.4
ACC_PDHC,N1-SY_	75.0	65.9	61.5	65.6	57.3	54.4
ACC_PDHC,N123-SY_	79.1	70.2	67.3	70.7	63.6	60.7

**Table 4:** Average scores for PSSUQ categories and technological skill levels.

Skill/ PSSUQ Category	System Usefulness Items 1–8	Information Quality Items 9–15	Interface Quality Items 16–18	Overall Score Item 1–18
None	4.7	4.9	5.4	4.9
Basic	5.4	5.3	5.8	5.4
Intermediate	6.1	6.6	6.7	6.5
Advanced	6.4	6.7	6.7	6.6
Total Average	5.4	5.6	5.9	5.5

## Supplementary Materials

The following are available online at https://www.mdpi.com/1424-8220/19/21/4764/s1, Table S1: List and meaning of Kinematic Parameters for each UPDRS task; Table S2: Items of the Post-Study System Usability Questionnaire (PSSUQ)
