# Supplementary material for: An Integrated Multi-Sensor Approach for the Remote Monitoring of Parkinson’s Disease
Source: Sensors (Basel). 2019 Nov 2;19(21):4764. doi: 10.3390/s19214764 (PMC6864792; doi:10.3390/s19214764)
Supplement: Supplementary file 1 [file sensors-19-04764-s001.pdf]

Table 1. List and Meaning of Kinematic Parameters for Unified Parkinson's Disease Rating Scale (UPDRS) tasks.

Task	Name	Meaning	Unit
FT	MO_m	Mean of Maximum Opening[2]	mm
	MOv	Variability[1] of Maximum Opening	-
	MOS_m	Mean of Maximum Speed (opening phase)	mm/s
	MOS_v	Variability[1] of Maximum Speed (opening phase)	-
	MCS_m	Mean of Maximum Speed (closing phase)	mm/s
	MCS_v	Variability[1] of Maximum Speed (closing phase)	-
	MA_m	Mean of Movement Amplitude[2]	mm
	MA_v	Variability[1] of Movement Amplitude	-
	Freq	Principal Frequency in voluntary movement band	Hz
	Dv	Variability[1] of Movement Duration	-
CO	MOS_m	Mean of Maximum Speed (opening phase)	mm/s
	MOS_v	Variability[1] of Maximum Speed (opening phase)	-
	MCS_m	Mean of Maximum Speed (closing phase)	mm/s
	MCS_v	Variability[1] of Maximum Speed (closing phase)	-
	MA_m	Mean of Movement Amplitude	mm
	MA_v	Variability[1] of Movement Amplitude	-
	D_m	Mean of Movement Duration	s
	D_v	Variability[1] of Movement Duration	-
PS	MR_m	Mean of Movement Rotation	deg
	MR_v	Variability[1] of Movement Rotation	-
	MSS_m	Mean of Maximum Speed (supination phase)	deg/s
	MSS_v	Variability[1] of Maximum Speed (supination phase)	-
	MPS_m	Mean of Maximum Speed (pronation phase)	deg/s
	MPS_v	Variability[1] of Maximum Speed (pronation phase)	-
	Freq	Principal Frequency in voluntary movement band	Hz
	DP_v	Variability[1] of Pronation Duration	-
LA	Θ_m	Mean of Angular Amplitude	deg
	Ω_m	Mean of Angular Speed	deg/s
	P_m	Mean of Pause	s
	R_m	Mean of Regularity	s
	F	Repetition Frequency	Hz
	$P_{X\Theta}$	Thigh Inclination Spectrum Power	-
	$P_{X\Omega}$	Thigh Angular Speed Spectrum Power	-
S2S	T	Total Duration	s
	T_f	Forwards Bending Duration	s
	T_b	Backwards Bending Duration	s
	D_t	Forwards/Backwards Duration Difference	s
	Θ	Average Bending Amplitude	deg
	Ω	Average Bending Speed	deg/s
G	GCT_m	Mean of Gait Cycle Time	s
	Limp	Limp	% of GCT
	SL	Stride Length	% of height
	$StepV_m$	Mean of Step Speed	%of height/s
	C	Cadence	steps/min
	RStep	Step Regularity	-
	S	Symmetry	-
	$ThighRoR_m$	Mean of Thigh Range of Rotation	deg

PSum	Total Spectrum Power	-

[1]: Variability is equivalent to the coefficient of variation CV, defined as the ratio of the standard deviation ϱ to the mean μ.

[2]: MOm indicates the maximum peak of fingers amplitude during the opening phase of the movement. MAm indicates the maximum excursion of the fingers (difference between maximum peak and minimum point in the same movement). MOm allows us to highlight anomalies during the opening phase; MAm anomalies during the closing phase.

Table 2. Items of the post-study system usability questionnaire (PSSUQ)[1].

Item #	Question
1.	Overall, I am satisfied with how easy it is to use the system
2.	It was simple to use the system
3.	I could effectively complete the tasks using the system
4.	I was able to complete the tasks quickly using the system
5.	I was able to efficiently complete the tasks using the system
6.	I felt comfortable using the system[2]
7.	It was easy to learn to use the system[2]
8.	I believe I could become efficient quickly using the system
9.	The system gave error messages that clearly told me how to recover
10.	Whenever I made a mistake using the system, I could recover easily and quickly
11.	The information provided by the system was clear
12.	It was easy to find the information I needed
13.	The information provided for the system was easy to understand
14.	The information was effective in helping me to complete the tasks
15.	The organization of information on the screen was clear
16.	The interface of this system was pleasant[2]
17.	I liked using the interface of the system[2]
18.	The system has all the functions I expected it to have
19.	Overall, I am satisfied with the system

[1]: Items were presented in Italian to Parkinson's disease (PD) participants. They have been translated only for publication readability. [2]: Items were also presented separately for the two subsystems (for upper and lower limbs).
